# Supplementary material for: MetaRibo-Seq measures translation in microbiomes
Source: Nat Commun. 2020 Jun 29;11:3268. doi: 10.1038/s41467-020-17081-z (PMC7324362; doi:10.1038/s41467-020-17081-z)
Supplement: Supplementary file 10 — Supplementary Data 7 [file 41467_2020_17081_MOESM10_ESM.zip › File2/Confidence_High_Taxonomy/306771_out.krona.html]

Javascript must be enabled to view this page.

members
magnitude
magnitudeUnassigned
count
unassigned
taxon
rank

306771\_out

27


SRS014923\_contig\_number\_contig-100\_10887.188501SRS020869\_contig\_number\_39401SRS043768\_contig\_number\_contig-100\_31635.133835SRS063190\_contig\_number\_contig-100\_25218.25219SRS104485\_contig\_number\_3964SRS142712\_contig\_number\_contig-100\_31998.31998SRS149075\_contig\_number\_contig-100\_29328.29329SRS893253\_contig\_number\_contig-100\_2613.25227SRS893358\_contig\_number\_contig-100\_17451.17452
2
superkingdom
9
27

976
1
phylum

1
200643
class

order
171549
1

family
815
1

816
1
genus

1
310297

SRS143598\_contig\_number\_1883
species

17
3
phylum
1239

SRS054956\_contig\_number\_15195SRS148511\_contig\_number\_contig-100\_2209.139605SRS893256\_contig\_number\_2504

class
186801
14

order
6
14

SRS019685\_contig\_number\_contig-100\_2577.233461SRS019787\_contig\_number\_7218SRS042284\_contig\_number\_contig-100\_2080.53541SRS050422\_contig\_number\_24531SRS893252\_contig\_number\_contig-100\_2387.120040SRS893378\_contig\_number\_contig-100\_7565.108733
186802

186803

SRS063040\_contig\_number\_993
1
4
family

genus
1
572511

2292967
1
species

SRS019267\_contig\_number\_8809

genus
2
33042

1896998
2
species

SRS076976\_contig\_number\_contig-100\_634.126009SRS147346\_contig\_number\_62380

family
31979
4

genus
3
1485

species

SRS019601\_contig\_number\_contig-100\_1538.170559SRS021219\_contig\_number\_contig-100\_935.84032SRS144537\_contig\_number\_41088
1720194
3

580596
1
genus

2292197
1

SRS013638\_contig\_number\_18396
species
